# Supplementary material for: Clustering and visualization of single-cell RNA-seq data using path metrics
Source: PLoS Comput Biol. 2024 May 29;20(5):e1012014. doi: 10.1371/journal.pcbi.1012014 (PMC11164391; doi:10.1371/journal.pcbi.1012014)
Supplement: S1 Text — (PDF) [file pcbi.1012014.s001.pdf]

1 Data Preprocessing

In this section the pre-processing of all RNA data sets is described. The main preprocessing steps are quality control, imputation with SAVER [1], and normalization. Below we provide information about quality control and imputation and then we describe how we used those steps according to the guidelines of each method.

1.1 Main steps

Quality Control: Quality control is applied on RNAmix1, RNAmix2, Cellmix, BaronPanc, PMC4K, Beta. Specifically, cells where at most 200 genes are expressed are filtered out. Also, only genes that are expressed in more than 3 cells are included in the data set. In addition, cells with percentage of expressed mitochondrial genes greater than 20% are excluded. The data sets TMpanc and TMLung as found in Figshare have passed a quality control check with cutoffs of at least 500 genes and 50,000 reads, so no additional filtering was applied.

Imputation: Imputation with SAVER [1] was applied to all RNA seq data sets apart from Cellmix. After removing multiplets the Cellmix data set included high quality data and every clustering method achieved high ARI, suggesting no need for further processing and imputation.

1.2 Preprocessing per method

Path metrics (PM), k-means, DBSCAN: After quality control and imputation, we normalize the data. RNAmix1, RNAmix2, TMLung, Beta, TMPanc, PBMC4K were row normalized and log transformed (data matrix had cells in rows and genes in columns). We then restrict to the top 2000 high variance genes. For the BaronPanc and CellMix, which have large sample size, SCT transformation was applied instead and the top 3000 variable genes were kept [2,3]. When needed, we rescale genes where variances were extremely high. As a next step we apply PCA for dimension reduction, keeping the top 40 PC's. Finally, denoising is applied by replacing each point with the mean of its local neighborhood, using a neighborhood size of $K = 12$ points. For very large data sets, one may want to use a larger K.

UMAP+DBSCAN, t-SNE+k-means: After quality control and imputation, we apply Linnnorm [4] to all data sets. Then, we restrict to the top 2000 high variance genes. When needed, we rescale genes with extremely high variance. Finally, we apply PCA for dimension reduction, keeping the top 40 PC's.

Seurat: For this method, we process the data as for PM and then use Seurat's [5] functions to find neighboring points and cluster them. Notice that here we adjust the parameter 'res', to retrieve the correct number of clusters.

Seurat_def: We follow the suggested processing and clustering workflow of Seurat [5] for all data sets. Notice that we normalize BaronPanc and CellMix with the SCT method [2,3]. Then data sets are clustered with adjusted resolution parameter, to retrieve the correct number of clusters.

SC3: After quality control and imputation we normalize the information of every cell and multiply by 10000. Then we use the log of the data for clustering with SC3 [6]. Exception to this are the BaronPanc and CellMix data set, for which we use SCT normalization.

scanpy: After quality control and imputation we use the lognormalization of scanpy [7]. Exception to this are the BaronPanc and CellMix data set, for which we use SCT

normalization.

RaceID3: We apply quality control on the cells of the counts of the data set. RaceID3 [8, 9] applies filtering and normalization in one step, which we adjust to have about the same amount of cells and genes as with other methods. Notice that we do not apply imputation because imputed data would not be counts, which are the required input of RaceID3.

SIMLR: For SIMLR [10] After quality control and imputation we normalize the information of every cell and multiply by 10000 and use the logorithm of those data. Exception to this are the BaronPanc and CellMix data set, for which we use SCT normalization.

1.3 Denoising with local averaging

As a denoising step, the scPMP algorithm performs local averaging. Specifically, the observed feature of a point is substituted for the average of that feature across the K_1 nearest neighbors of the point. The effect of local averaging on clustering with scPMP was explored, and it was found that local averaging not only contributes to an increase in ARI, but it also reduces the overall runtime of the algorithm (Table A).

Furthermore, we explore the runtime and clustering performance of scPMP when coupled with other preprocessing methods than those mentioned in section 1.2. In more detail, we use the normalization and dimension reduction process of scVI [11], which assumes that expression data are generated from a Zero Inflated Negative Binomial (ZINB) model and estimates the parameters of this model by applying variational inference. We additionally investigate the effect of scVI preprocessing on scPMP clustering with and without local averaging. According to our results in Table B, scVI yielded better ARI values when combined with local averaging for most cases (Baron(p=2), PBMC4K(p=2), and didn't outperform scPMP clustering with the default preprocessing and denoising methods (as seen in the last two rows of Table A).

Table A. Clustering performance with and without local averaging denoising.

Dataset	Local Averaging	ARI	ECP	ECA	complete runtime	clustering runtime
BaronPanc	false	0.791	0.289	0.158	4.643	4.197
PBMC4k	false	0.744	0.321	0.196	18.077	12.844
BaronPanc	true	**0.969**	0.081	0.077	0.739	**0.682**
PBMC4k	true	**0.978**	0.055	0.096	4.936	**4.702**

Table B. Clustering performance after preprocessing with scVI.

Dataset	Local Averaging	ARI	ECP	ECA	complete runtime	clustering runtime
BaronPanc(p=2)	false	0.653	0.440	0.332	2.436	2.300
BaronPanc(p=2)	true	0.721	0.373	0.277	2.386	2.217
PBMC4k(p=4)	false	0.745	0.324	0.201	12.550	12.132
PBMC4k(p=4)	true	0.741	0.320	0.188	11.109	10.745
PBMC4k(p=2)	false	0.744	0.319	0.191	13.475	13.072
PBMC4k(p=2)	true	0.748	0.315	0.190	12.732	12.348

References

1. Huang, M., Wang, J., Torre, E., Dueck, H., Shaffer, S., Bonasio, R., Murray, J., Raj, A., Li, M. & Zhang, N. SAVER: gene expression recovery for single-cell RNA

sequencing. *Nature Methods*. **15**, 539-542 (2018)

2. Hafemeister, C. & Satija, R. Normalization and variance stabilization of single-cell RNA-seq data using regularized negative binomial regression. *Genome Biology*. **20** (2019)

3. Choudhary, S. & Satija, R. Comparison and evaluation of statistical error models for scRNA-seq. *Genome Biology*. **23** (2022)

4. Yip, S., Wang, P., Kocher, J., Sham, P. & Wang, J. Linnorm: improved statistical analysis for single cell RNA-seq expression data. *Nucleic Acids Research*. **45**, e179-e179 (2017,9), https://doi.org/10.1093/nar/gkx828

5. Stuart, T., Butler, A., Hoffman, P., Hafemeister, C., Papalexi, E., III, W., Hao, Y., Stoeckius, M., Smibert, P. & Satija, R. Comprehensive Integration of Single-Cell Data. *Cell*. **177** pp. 1888-1902 (2019), https://doi.org/10.1016/j.cell.2019.05.031

6. Kiselev, V., Kirschner, K., Schaub, M., Andrews, T., Yiu, A., Chandra, T., Natarajan, K., Reik, W., Barahona, M., Green, A. & Hemberg, M. SC3: consensus clustering of single-cell RNA-seq data. *Nature Methods*. **14** pp. 483-486 (2017,5), https://doi.org/10.1038/nmeth.4236

7. Wolf, F., Angerer, P. & Theis, F. SCANPY: large-scale single-cell gene expression data analysis. *Genome Biology*. **19** (2018,2),

8. Grün, D. & Others Revealing Dynamics of Gene Expression Variability in Cell State Space.. *Nature Methods*. **17** pp. 45-49 (2018)

9. Herman, J., Grün, D. & Others FateID infers cell fate bias in multipotent progenitors from single-cell RNA-seq data. *Nature Methods*. **15**, 379 (2018)

10. Wang, B., Zhu, J., Pierson, E., Ramazzotti, D. & Batzoglou, S. Visualization and analysis of single-cell RNA-seq data by kernel-based similarity learning. *Nature Methods*. **14** pp. 414-416 (2017,4), https://doi.org/10.1038/nmeth.4207

11. Lopez, R., Regier, J., Cole, M., Jordan, M. & Yosef, N. Deep generative modeling for single-cell transcriptomics. *Nature Methods*. **15**, 1053-1058 (2018)
