# Supplementary material for: Clustering and visualization of single-cell RNA-seq data using path metrics
Source: PLoS Comput Biol. 2024 May 29;20(5):e1012014. doi: 10.1371/journal.pcbi.1012014 (PMC11164391; doi:10.1371/journal.pcbi.1012014)
Supplement: S2 Text — (PDF) [file pcbi.1012014.s002.pdf]

1 Additional Clustering Results

Here we present more clustering evaluation results based on Entropy of Cluster Accuracy (ECA) and Entropy of cluster Purity (ECP). The ECA can quantify the variety of true labels within a predicted cluster and ECP can quantify the variety of predicted cluster labels within a true group.

Definition 1 *Let N represent the number of true groups and M the number of predicted clusters. Let N_j be the number of true groups with data points within the jth predicted cluster and similarly let M_j be the number of predicted clusters with data points within the jth true group. Finally let $p(x_j)$ denote the proportion of data points belonging to the jth true group that are within a given j^{th} predicted cluster and let $p_i(y_j)$ denote the proportion of data points of jth predicted cluster that are within a given i^{th} true group. Then:*

$$\mathrm{ECA} = -\frac{1}{M}\sum_{i=1}^{M}\sum_{j=1}^{N_i} p_i(x_j)\log(p(x_j)),$$

$$\mathrm{ECP} = -\frac{1}{N}\sum_{i=1}^{N}\sum_{j=1}^{M_i} p_i(y_j)\log(p(y_j)).$$

For a given clustering, low ECA means that data points in a predicted cluster originate from the same true group. On the other hand, low ECP indicates that almost all the data points in a true group were assigned the same clustering label. Use of ECP and ECA in clustering of scRNAseq data was also found in [1].

Table A. ECP for manifold data.

Method	Balls	EWB	Swiss	SO(3)
k-means	**0.082**	1.050	0.588	1.084
DBSCAN	0.385	0.114	**0**	**0**
UMAP+DBSCAN	0.941	0.695	**0**	**0**
t-SNE+ k-means	0.153	0.630	**0**	0.440
Seurat	0.255	0.193	**0**	**0**
$\mathrm{PM}_{1.5}$	0.123	0.447	**0**	0.460
PM_2	0.142	**0.020**	**0**	**0**
PM_4	0.253	0.268	**0**	**0**

Table B. ECA for manifold data.

Method	Balls	EWB	Swiss	SO(3)
k-means	**0.082**	1.096	0.633	1.089
DBSCAN	0.362	0.231	**0**	**0**
UMAP+DBSCAN	0.200	**0.014**	**0**	**0**
t-SNE+ k-means	0.147	0.582	**0**	0.440
Seurat	0.250	0.183	**0**	**0**
$\mathrm{PM}_{1.5}$	0.120	0.461	**0**	0.462
PM_2	0.138	0.020	**0**	**0**
PM_4	0.248	0.291	**0**	**0**

Table C. ECP for RNA data.

Method	RNA1	RNA2	TMLung	Beta	TMPanc	BaronPanc	PBMC4k	CellMix
SC3	0.328	0.114	0.294	0.058	0.070	0.301	0.062	0
Scanpy	0.517	0.183	0.322	0.128	0.516	0.088	0.057	0
RaceID3	0.381	0.665	**0.182**	0.351	0.268	0.413	0.310	0
SIMLR	0.151	0.267	0.275	0.048	0.543	0.380	0.360	0
Seurat	0.292	0.282	0.230	0.155	0.540	0.122	0.053	0.027
Seurat_def	0.320	0.258	0.436	0.114	0.284	0.089	0.062	0
k-means	0.131	0.255	0.244	0.058	0.215	0.395	0.316	0
DBSCAN	**0.075**	0.141	0.404	0.083	0.138	0.109	**0.051**	0
UMAP+db	0.151	0.226	0.413	**0.023**	**0.061**	0.248	0.087	0
t-SNE+ k-means	0.102	0.133	0.437	0.052	0.494	0.402	0.451	0.147
$PM_{1.5}$	0.096	0.136	0.197	0.058	0.482	0.273	0.312	0
PM_2	0.096	**0.062**	0.323	0.058	0.141	**0.081**	0.308	**0**
PM_4	0.096	0.114	0.184	0.108	0.260	0.226	0.055	0

Table D. ECA for RNA data.

Method	RNA1	RNA2	TMLung	Beta	TMPanc	BaronPanc	PBMC4k	CellMix
SC3	0.289	0.114	0.228	0.058	0.132	0.368	0.328	0
Scanpy	0.481	0.242	0.314	0.129	0.309	0.093	**0.054**	0
RaceID3	0.336	0.621	**0.168**	0.342	**0.122**	0.181	0.207	0.000
SIMLR	0.163	0.294	0.263	0.049	0.407	0.104	0.190	0
Seurat	0.319	0.230	0.193	0.153	0.290	0.097	0.265	0
Seurat_def	0.256	0.270	0.423	0.109	0.289	0.112	0.106	0
k-means	0.147	0.268	0.221	0.058	0.194	0.164	0.193	0
DBSCAN	0.090	0.188	0.368	0.465	0.202	0.146	0.262	0
UMAP+db	**0.078**	0.151	0.449	0.364	0.124	**0.076**	0.163	0
t-SNE+ k-means	0.104	0.137	0.426	**0.052**	0.259	0.171	0.187	0.126
$PM_{1.5}$	0.110	0.146	0.180	0.058	0.305	0.147	0.196	0
PM_2	0.110	**0.071**	0.362	0.058	0.196	0.077	0.195	**0**
PM_4	0.110	0.123	0.230	0.106	0.156	0.159	0.096	0

Table E. Downsampling results.

Dataset	Seurat	Seurat_def	$PM_{1.5}$	PM_2	PM_4
100% of Baron's Pancreatic	0.941	0.971	0.804	0.969	0.853
50% of Baron's Pancreatic	0.880	0.844	0.969	0.969	0.969
25% of Baron's Pancreatic	0.973	0.705	0.973	0.973	0.973
10% of Baron's Pancreatic	0.410	0.185	0.674	0.939*	0.804

Table F. Predicted number of clusters for Seurat and Path metrics for RNA data.

Method	RNA1	RNA2	TMLung	Beta	TMPanc	BaronPanc	PBMC4k	CellMix
Seurat_res=0.8	**7**	8	**7**	6	11	12	13	14
$PM_{1.5}$	12	11	8	4	15	9	**4**	**5**
PM_2	**7**	**7**	9	4	5	**8**	5	**5**
PM_4	8	8	16	4	5	7	**4**	**5**
True k	7	7	7	3	7	8	4	5

k is the true number of clusters.

References

1. Tian L, Dong X, Freytag S, Lê Cao KA, Su S, JalalAbadi A, et al. Benchmarking single cell RNA-sequencing analysis pipelines using mixture control experiments. Nature methods. 2019;16(6):479–487.
