# Supplementary material for: Clustering and visualization of single-cell RNA-seq data using path metrics
Source: PLoS Comput Biol. 2024 May 29;20(5):e1012014. doi: 10.1371/journal.pcbi.1012014 (PMC11164391; doi:10.1371/journal.pcbi.1012014)
Supplement: S3 Text — (PDF) [file pcbi.1012014.s003.pdf]

# 1 Clustering visualizations on PCA and scPMP embedding

Fig A shows the PCA and $PM_2$ plots (uniform sampling was performed in each coordinate). Note the PCA plot is elongated due to the approximate orthogonality, but the PM embedding exaggerates this effect by stretching the data in regions of high density (i.e. "useful" directions), and shrinking in noisy directions where the data is sparse. Thus the clusters in the $PM_2$ plot appear even more elongated. However, the bar-like appearance is not indicative of the true shape of the clusters but an artifact of the projection into the lower-dimensional space. Fig B, C, D show the scPMP embeddings of our benchmarking data sets colored by both the ground truth and predicted labels. For many of the data sets, some clusters appear as elongated bars in the $PM_2$ embedding. This tends to occur when clusters live in nearly orthogonal spaces due to different genetic signatures, i.e. they have high/low expression values on disjoint sets of genes. This effect also occurs for PCA, but the denoising effect of the $PM_2$ embedding exaggerates the effect. Consider for example 3 clusters, where $C_1$ expresses high values of $x_1, x_2$, $C_2$ expresses high values of $x_3, x_4$, and $C_3$ expresses high values of $x_5, x_6$, but all three clusters have "noise" appearing in all dimensions.

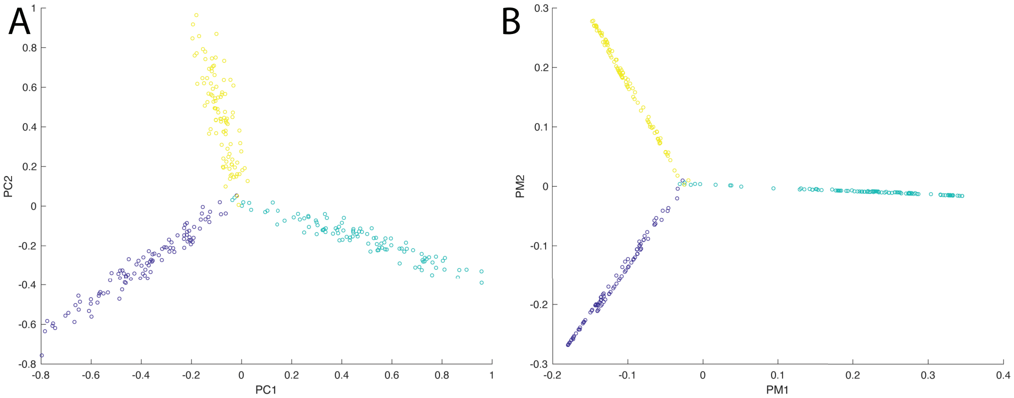


**Fig A. Elongation effect for PCA and $PM_2$ embeddings.**

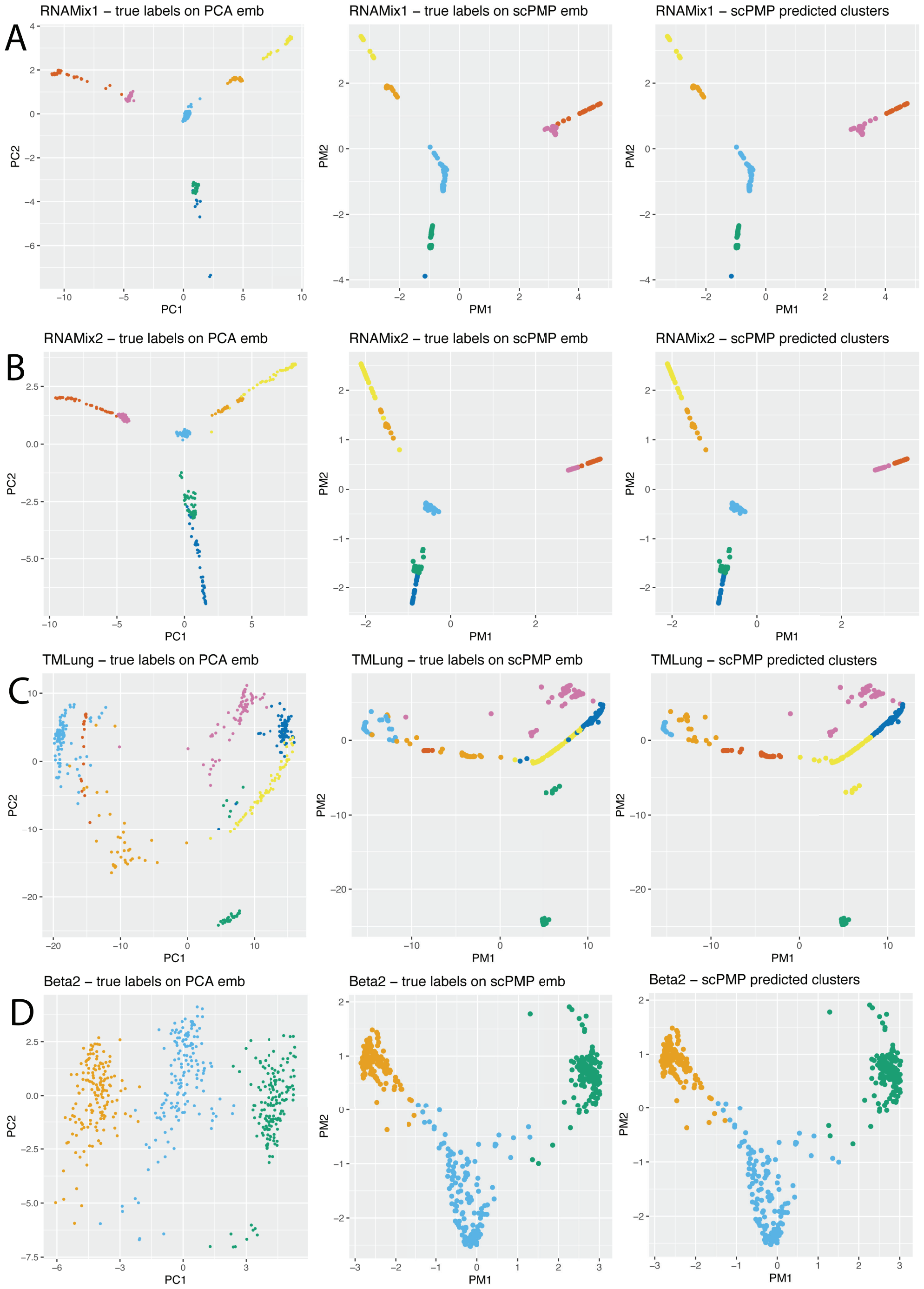


**Fig B. Clustering results on PCA and scPMP embedding for RNAmix1, RNAmix2, TMLung, and Beta.**

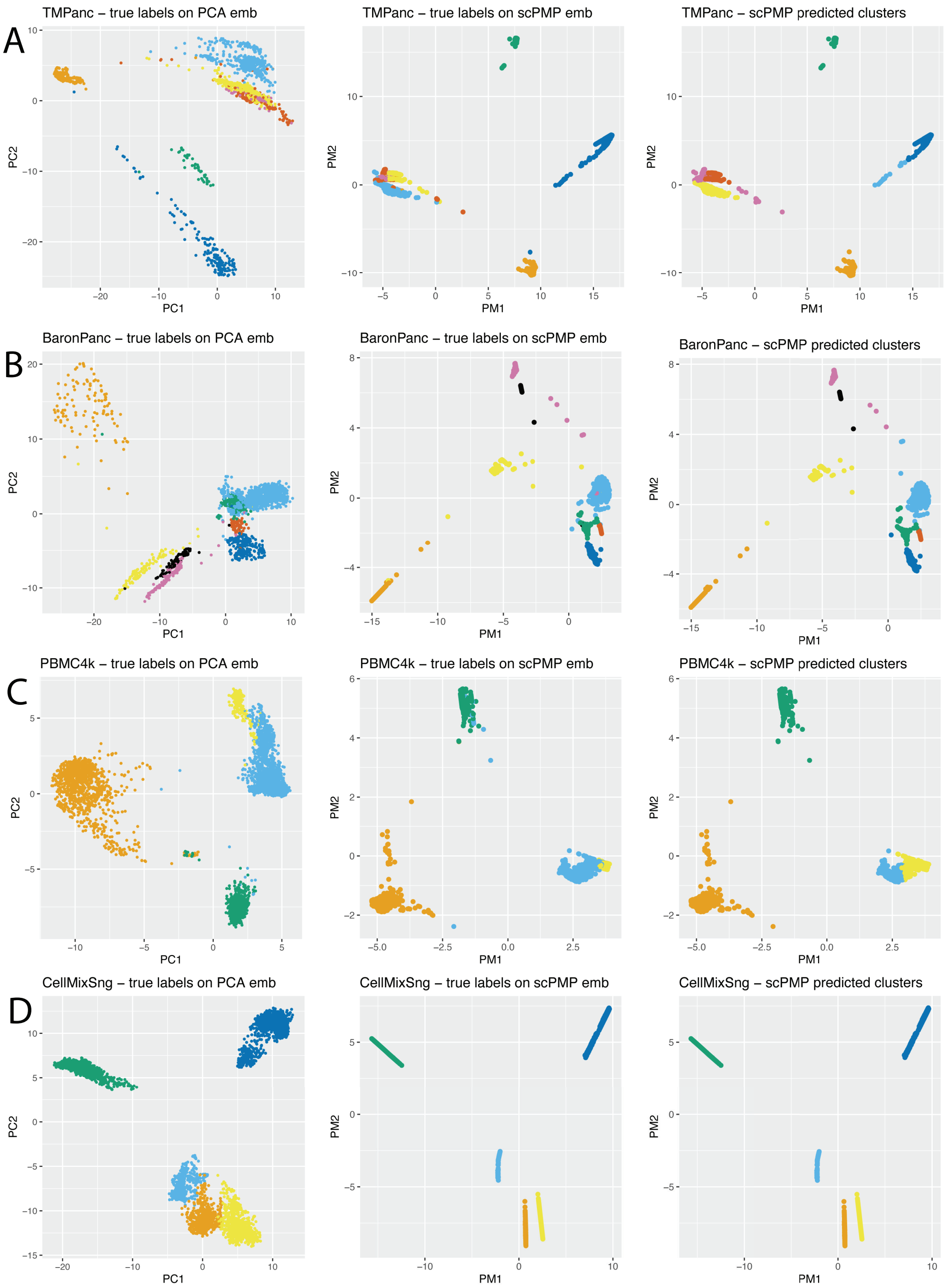


**Fig C. Clustering results on PCA and scPMP embedding for TMPanc, BaronPanc, PBMC4K, and CellMix.**

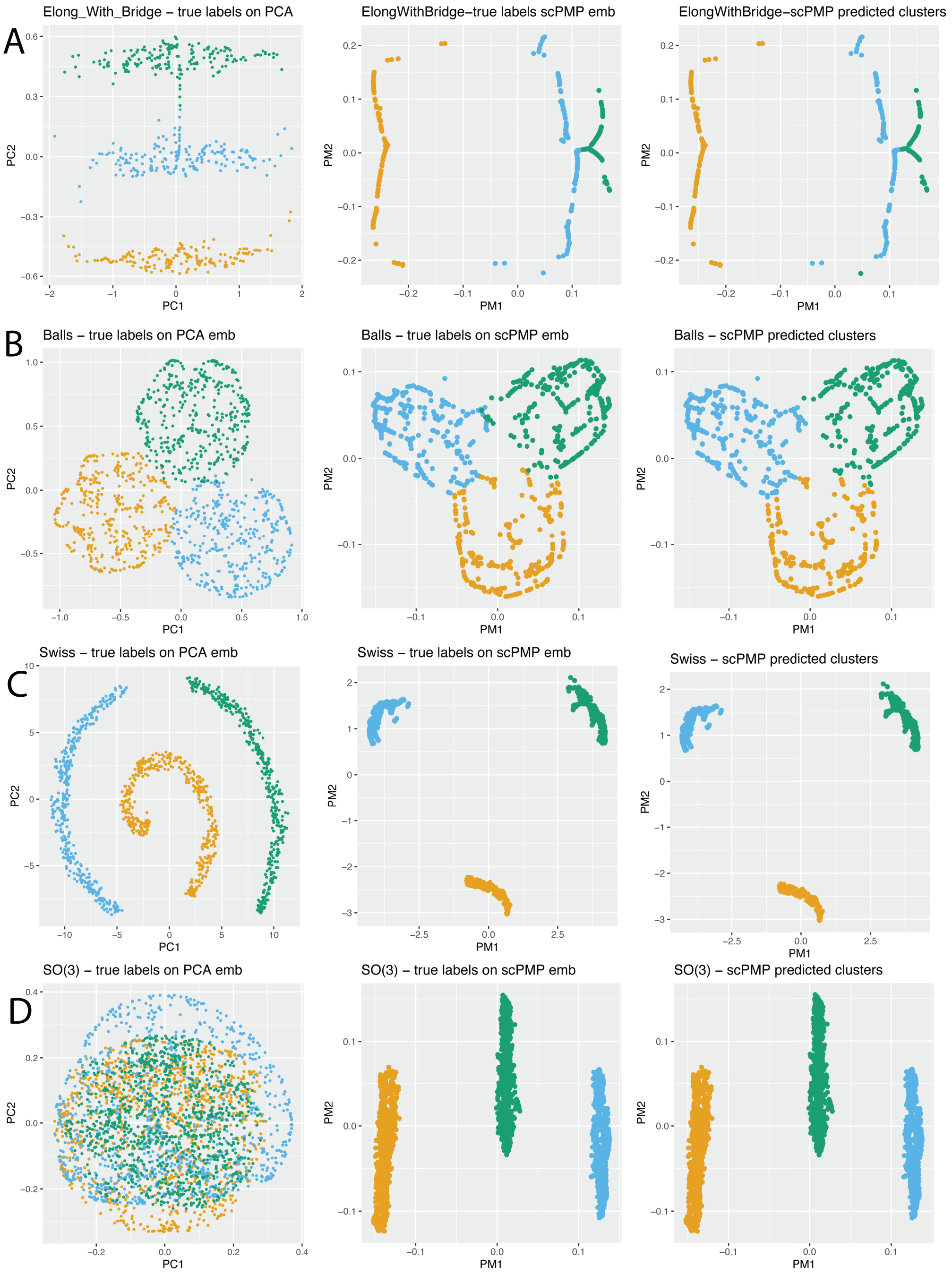


**Fig D. Clustering results on PCA and scPMP embedding for Elongated with Bridge, Balls, Swiss, and SO(3).**
